# Supplementary material for: The female protective effect against autism spectrum disorder
Source: Cell Genom. 2022 Jun 8;2(6):100134. doi: 10.1016/j.xgen.2022.100134 (PMC9903803; doi:10.1016/j.xgen.2022.100134)
Supplement: Document S1. Figures S1–S3, Tables S1–S8, and Methods S1 and S2 [file mmc1.pdf]

Cell Genomics, Volume *2*

Supplemental information

The female protective effect

against autism spectrum disorder

Emilie M. Wigdor, Daniel J. Weiner, Jakob Grove, Jack M. Fu, Wesley K. Thompson, Caitlin E. Carey, Nikolas Baya, Celia van der Merwe, Raymond K. Walters, F. Kyle Satterstrom, Duncan S. Palmer, Anders Rosengren, Jonas Bybjerg-Grauholm, iPSYCH Consortium, David M. Hougaard, Preben Bo Mortensen, Mark J. Daly, Michael E. Talkowski, Stephan J. Sanders, Somer L. Bishop, Anders D. Børglum, and Elise B. Robinson

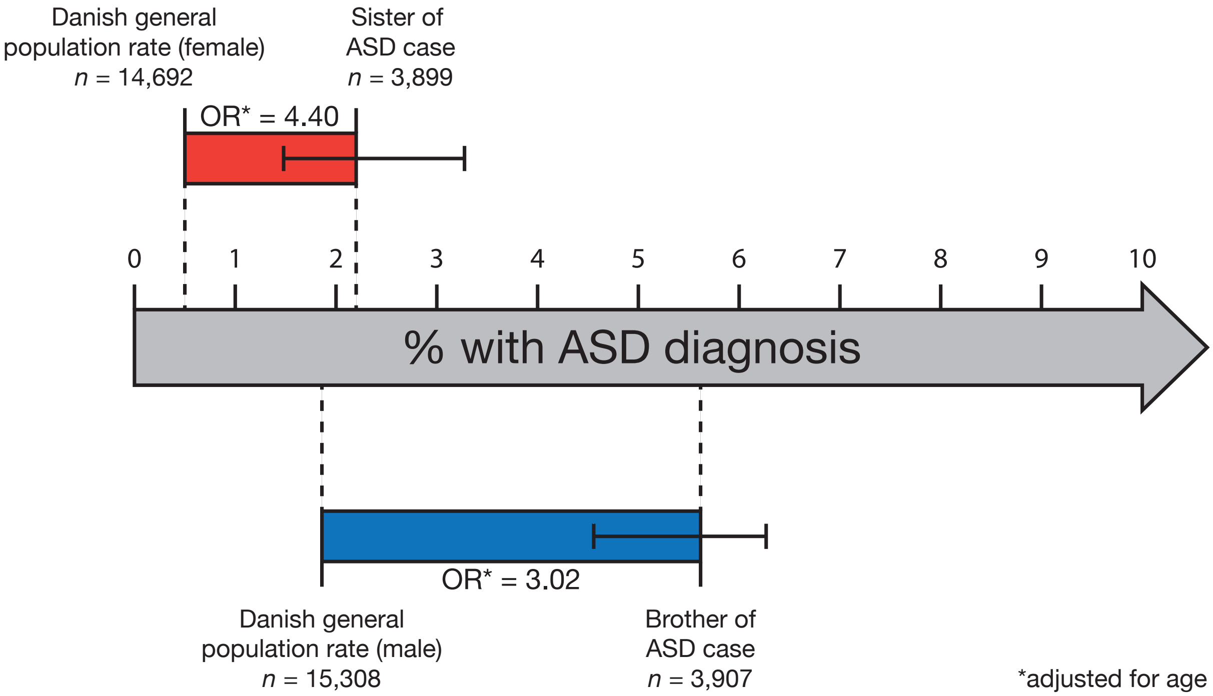

Figure S1. Increased risk for ASD in sisters and brothers of ASD cases compared to Danish population controls. ORs are the exponentiated betas from logistic regression (see STAR Methods; Sibling recurrence of ASD and ID). Error bars are 95% confidence intervals. The start positions of the colored bars represent the prevalence of ASD in the Danish general population, by sex. The end positions of the colored bars represent the projected risk of ASD in siblings, by sex. The end positions are calculated by multiplying the baseline prevalence by the OR.

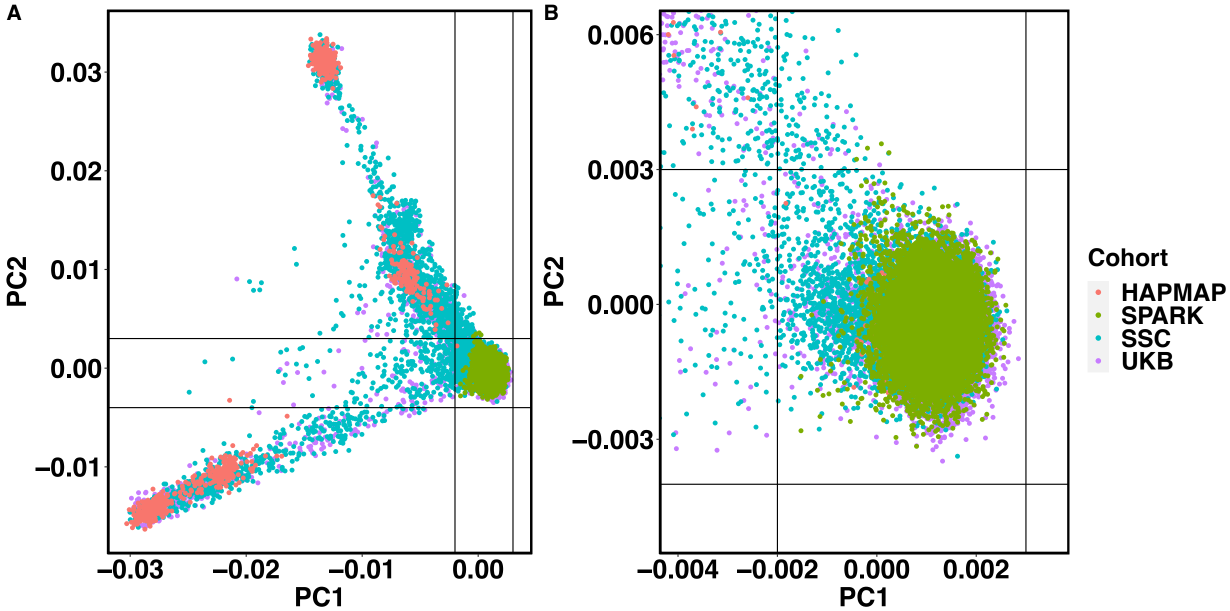

Figure S2. PCA of SPARK, SSC and UKB with HapMap. Colored dots represent individuals from HapMap, SPARK, SSC and UKB (*n*=48,159) (see STAR Methods; Ancestry definition in SSC, SPARK and UKB). A) All 48,159 samples plotted for principal component 1 and principal component 2. B) A selected sub-sample of our cases and controls that clustered with Europeans in HapMap (-0.002 < PC1 < 0.003, -0.004 < PC2 < 0.003). Horizontal and vertical lines correspond to those PC thresholds.

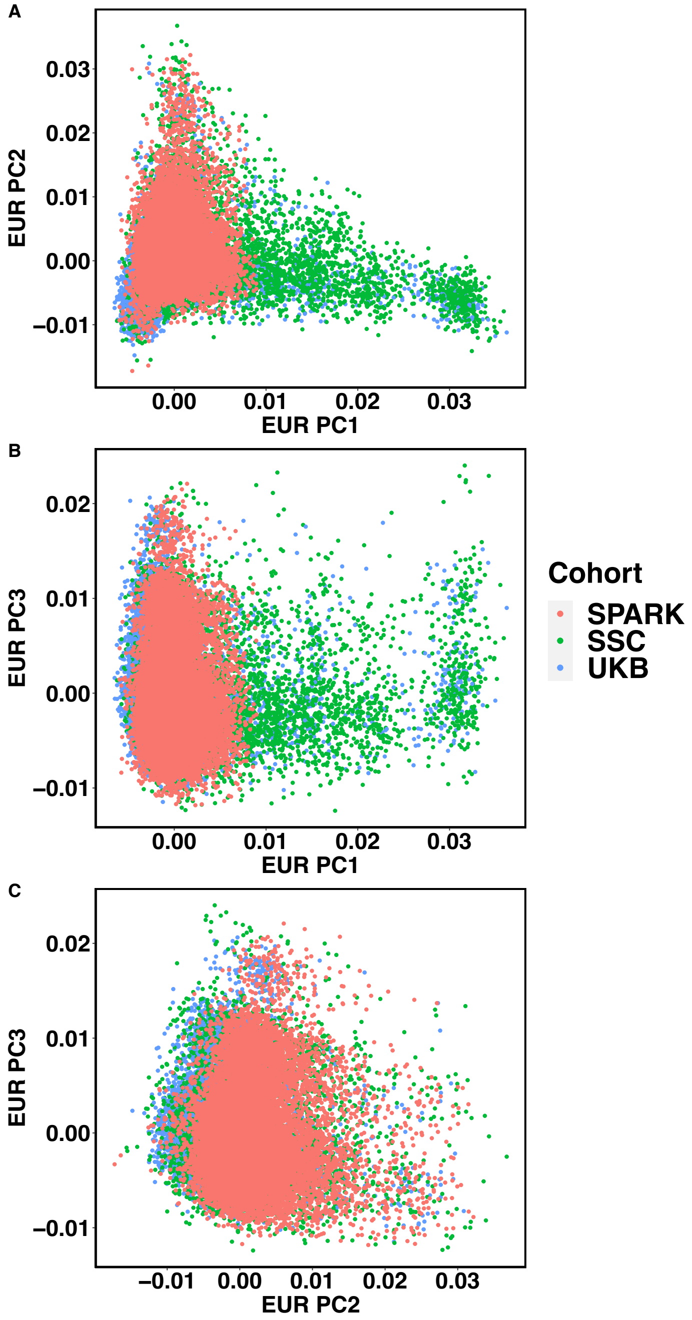

Figure S3. Within-European PCA of SPARK, SSC and UKB. Principal components in the European ancestry subset of UKB, SSC and SPARK defined in Supplementary Figure 2 (see STAR Methods; Ancestry definition in SSC, SPARK and UKB). A) Principal component 1 versus principal component 2. B) Principal component 1 versus principal component 3. C) Principal component 2 versus principal component 3.

Phenotype	OR siblings of female cases (95% CI) (N=1,707 siblings, N=3,414 controls)	OR siblings of male cases (95% CI) (N=6,270 siblings, N=12,540 controls)	Wald test p value
ID without ASD	1.77 (0.88-3.56)	1.71 (1.13-2.60)	8.88×10^{-1}
ASD and ID	6.06 (2.40-15.28)	4.66 (2.72-7.98)	1.10×10^{-2}
ASD without ID	7.19 (5.09-10.09)	3.76 (3.10-4.54)	$P < 1.0 \times 10^{-10}$

Table S1. Contains data underlying Figure 1. Siblings of cases with diagnosis of ASD without ID.

Phenotype	OR siblings of female cases (95% CI) (N=506 siblings, 1,012 controls)	OR siblings of male cases (95% CI) (N=811 siblings, N=1,622 controls)	Wald test p value
ID without ASD	10.03 (3.80-26.44)	11.03 (4.89-24.85)	1.21×10^{-1}
ASD and ID	2.00 (0.12-32.07)	6.02 (0.63-57.95)	2.80×10^{-2}
ASD without ID	2.01 (0.80-5.12)	1.49 (0.79-2.80)	3.60×10^{-1}

Table S2. Contains data underlying Figure 1. Siblings of cases with diagnosis of ID without ASD.

Group	N mothers	N fathers	Beta raw	SE raw	P value	Beta scaled	SE scaled
SSC	2061	2079	8.40×10^{-9}	3.16×10^{-9}	0.00798	8.29×10^{-2}	3.12×10^{-2}
SPARK	5375	3847	8.66×10^{-9}	2.14×10^{-9}	5.21×10^{-5}	8.55×10^{-2}	2.11×10^{-2}
SSC+SPARK	7436	5926	8.77×10^{-9}	1.77×10^{-9}	6.95×10^{-7}	8.66×10^{-2}	1.75×10^{-2}

Table S3. Contains data underlying Figure 2. Results of linear regression of PRS ~ Mother (1/0) + PC1-15 in SSC, SPARK and SSC+SPARK.

Group	N mothers	N fathers	N UKB	Beta raw	SE raw	P value	Beta scaled	SE scaled
UKB, SSC+SPARK	NA	5926	18862	1.76×10^{-8}	1.66×10^{-9}	2.04×10^{-26}	1.74×10^{-1}	1.63×10^{-2}
UKB, SSC+SPARK	7436	NA	18862	2.68×10^{-8}	1.52×10^{-9}	4.87×10^{-69}	2.64×10^{-1}	1.50×10^{-2}
UKB, SSC+SPARK	7436	5926	18862	2.33×10^{-8}	1.27×10^{-9}	1.93×10^{-75}	2.30×10^{-1}	1.25×10^{-2}

Table S4. Contains data underlying Figure 2. Results of linear regression of PRS ~ Parent (1/0) + PC1-15 in UKB and SSC+SPARK.

Group	N fathers	N mothers	Beta raw	SE raw	P value	Beta scaled	SE scaled
UKB	8679	10183	2.10×10^{-9}	1.48×10^{-9}	1.54×10^{-1}	2.08×10^{-2}	1.46×10^{-2}

Table S5. Contains data underlying Figure 2. Results of linear regression of PRS ~ Mother (1/0) + PC1-15 in UK Biobank.

Group	N mothers	N probands	Beta raw	SE raw	P value	Beta scaled	SE scaled
SSC+SPARK	7436	7628	9.35×10^{-9}	1.64×10^{-9}	1.22×10^{-8}	9.23×10^{-2}	1.62×10^{-2}

Table S6. Contains data underlying Figure 2. Results of linear regression of PRS ~ Proband (1/0) + PC1-15.

Group	N	Raw mean	Raw lower 95 % CI	Raw upper 95 % CI	P value	Scaled mean	Scaled lower 95 % CI	Scaled upper 95 % CI
Male proband, de novo	436	5.90×10^{-9}	-1.04×10^{-9}	1.28×10^{-8}	0.10	8.13×10^{-2}	-1.43×10^{-2}	1.77×10^{-1}
Male proband, no de novo	3468	1.26×10^{-8}	1.02×10^{-8}	1.49×10^{-8}	9.72×10^{-25}	1.73×10^{-1}	1.40×10^{-1}	2.06×10^{-1}
Female proband, de novo	159	8.16×10^{-9}	-2.64×10^{-9}	1.90×10^{-8}	0.14	1.13×10^{-1}	-3.64×10^{-2}	2.61×10^{-1}
Female proband, no de novo	757	1.64×10^{-8}	1.15×10^{-8}	2.13×10^{-8}	7.82×10^{-11}	2.26×10^{-1}	1.59×10^{-1}	2.94×10^{-1}
Male siblings	1519	-3.89×10^{-9}	-7.45×10^{-9}	-3.31×10^{-10}	0.032	-5.37×10^{-2}	-1.03×10^{-1}	-4.57×10^{-3}
Female siblings	1611	-1.53×10^{-9}	-5.02×10^{-9}	1.95×10^{-9}	0.39	-2.11×10^{-2}	-6.92×10^{-2}	2.70×10^{-2}
Mothers	4820	4.53×10^{-9}	2.54×10^{-9}	6.51×10^{-9}	8.20×10^{-6}	6.24×10^{-2}	3.50×10^{-2}	8.98×10^{-2}
Fathers	4820	-4.53×10^{-9}	-6.51×10^{-9}	-2.54×10^{-9}	8.20×10^{-6}	-6.24×10^{-2}	-8.98×10^{-2}	-3.50×10^{-2}

Table S7. Contains data underlying Figure 3. pTDT results for SSC and SPARK jointly.

Population Prevalence of ASD among female population controls (N=14,692)	Population Prevalence of ASD among male population controls (N=15,308)	OR (95% CI) Sisters (N=3,899), Controls (N=7,798)	OR (95% CI) Brothers (N=3,907), Controls (N=7,814)	Wald p value
0.5%	1.86%	4.40 (2.96-6.55)	3.02 (2.45-3.73)	1.75×10^{-9}

Table S8. Contains data underlying Figure S1. Sisters and brothers of cases with diagnosis of ASD (See Methods S1; STAR Methods: Sibling recurrence of ASD and ID).

Methods S1. Sibling recurrence of ASD and ID, by sibling sex. Contains additional methods details from STAR Methods: Sibling recurrence of ASD and ID.

We hypothesized that given a FPE, brothers of ASD cases would have increased risk for ASD compared to sisters of ASD cases. Sisters of ASD cases have significantly increased risk for ASD (OR = 4.40, 95% CI = 2.96-6.55), calculated as fold-change over age and sex matched controls, compared to brothers of ASD cases (OR = 3.02, 95% CI = 2.45-3.73, $P = 1.75 \times 10^{-9}$, Wald test; see STAR Methods: Siblings recurrence of ASD and ID). However, the baseline prevalence of ASD amongst females in the Danish general population is lower than the baseline prevalence of ASD amongst males in the Danish general population. Therefore, sisters of ASD cases' overall risk for ASD remains lower than for brothers of ASD cases. The prevalence of ASD in the female Danish general population is 0.5%. A 4.4 fold increase in risk for ASD with a baseline risk of 0.5% would result in a 2.2% chance of having ASD. The prevalence of ASD in the male Danish general population is 1.86%. A 3.02 fold increase in risk for ASD with a baseline risk of 1.86% would result in a 5.62% chance of having ASD.

For each family, we selected an index ASD case regardless of sex and comorbid ID status. For each index case, we randomly selected a sibling, each with equal probability of selection. We then split the selected siblings by sex, into sisters and brothers of ASD cases.

Selected siblings were subset to those born between 1981 and 2005. Each of these siblings were matched with two age and sex matched Danish population representative controls (*n* = 30,000). All siblings of index cases were removed from the control cohort before being matched.

We then ran logistic regression, $NDD\ case\ status \sim 1_{sib\ of\ case}$ (where $1_{sib\ of\ case}$ is an indicator variable for whether the individual was the sibling of an NDD case ($= 1$), or an age and sex matched control ($= 0$)), for sisters and brothers separately to investigate whether they have an increased risk for *ASDnoD*, *ASDandID*, and *IDnoASD* compared to age and sex matched controls.

ORs for increased risk with sibling case status are the exponentiated effect sizes for the association between sibling case status and diagnosis of a psychiatric disorder. To compare ORs between sisters and brothers of ASD cases, we conducted a Wald test.

Methods S2: SPARK ancestry assignment, pre-imputation quality control and imputation. Contains additional methods details from STAR Methods: SPARK Imputation.

<u>Ancestry Assignment</u>

Self-reported demographic data were not available for the majority of SPARK participants, though existing data suggests that the racial and ethnic representation approximates that of the larger US population.[27,28] To determine which individuals were of European ancestry, we first restricted to a maximally unrelated ($\hat{\pi}$ < 0.09375; midpoint between 3rd and 4th degree relatives) set of pedigree-reported founders as defined by PRIMUS (*n* = 13,976)[59]. We then performed[60] PCA via EIGENSOFT[38,39] on this sample after combining with those in the Human Genome Diversity Project (HGDP).[60–63] We used the HGDP sample in order to capture the full axes of ancestral variation within the SPARK sample. For the purposes of PCA, only variants passing a strict set of

Ricopili QC measures (missingness < 5%, HWE $P > 1.0 \times 10^{-3}$, strand-unambiguous, and not in regions of high LD such as the MHC and chr8 inversion) were used, pruned to be pairwise independent at $r^2 < 0.2$. Additionally, 70 SNPs with allele frequency differences of > 0.2 between SPARK and HGDP self-reported EUR samples were removed. Non-founders were projected into the PC space of unrelated founders and the HGDP sample using hwe_normalized_pca in Hail (https://hail.is/). ADMIXTURE[36] was used in order to identify ancestral subpopulations within the joint SPARK + HGDP sample described above; cross-validation suggested the presence of 5 subpopulations. Individuals were labelled as having primarily EUR ancestry (n = 17,098) if their ancestral makeup, as determined by ADMIXTURE, was 85% or greater from Population 0. Population 0 was determined to be the EUR subpopulation as it contained a high prevalence of HGDP EUR and self-reported SPARK White/Caucasian relative to other HGDP or other self-reported ancestry, respectively.

Pre-imputation QC

Upon restricting to individuals of primarily EUR ancestry, we undertook both sample and variant-level QC procedures consistent with the Ricopili and picopili standards. Samples were removed for the following reasons: missingness rate > 0.02 (n = 71), absolute F_{HET} homozygosity rate > 0.2 (n = 2), Mendelian error rate > 0.02 (n = 0), sex check errors (n = 14), and cryptic relatedness ($\hat{\pi} > 0.09375$ across families; n = 46). All self-reported pedigrees were confirmed via genetically derived kinship coefficients. Variants retained for inclusion were required to have missingness < 0.02; absolute differential missingness between cases and controls < 0.02; Mendelian error rates < 0.01; and HWE $P > 1.0 \times 10^{-10}$ in founder cases, HWE $P > 1.0 \times 10^{-6}$ in founder controls, and HWE $P > 1.0 \times 10^{-10}$ in all founders. Post-QC, 16,965 samples and 557,368 variants remained for imputation.

Imputation

Autosomes were imputed to the Haplotype Reference Consortium (HRC)[52] reference panel using SHAPEIT[48] and IMPUTE2[40,48] in the picopili pipeline (https://github.com/Nealelab/picopili). Phasing was performed using SHAPEIT including its duoHMM algorithm, which uses pedigree information when available for more accurate results.[64] Best-guess genotypes were called for autosomal SNPs (minimum posterior probability > 0.8) and subsequently filtered to SNPs with missingness < 0.02, INFO > 0.6, and MAF > 0.005, for a final total of 7,124,628 SNPs with a genotyping rate of 0.995 across 16,965 samples.
